# Patient-Reported Outcome Measure for Real-time Symptom Assessment in Women With Endometriosis: Focus Group Study

**DOI:** 10.2196/28782

**Published:** 2021-12-03

**Authors:** Esther van Barneveld, Arianne Lim, Nehalennia van Hanegem, Lisa Vork, Alexandra Herrewegh, Mikal van Poll, Jessica Manders, Frits van Osch, Wilbert Spaans, Gommert van Koeveringe, Desiree Vrijens, Joanna Kruimel, Marlies Bongers, Carsten Leue

**Affiliations:** 1 Department of Gynaecology and Obstetrics Maastricht University Medical Centre+ Maastricht Netherlands; 2 Grow School of Oncology and Developmental Biology, Maastricht University Maastricht Netherlands; 3 Department of Gynaecology and Obstetrics, University Medical Centre Utrecht Utrecht Netherlands; 4 Department of Internal Medicine, Division of Gastroenterology and Hepatology, Maastricht University Medical Centre+ Maastricht Netherlands; 5 NUTRIM School of Nutrition and Translational Research in Metabolism, Maastricht University Maastricht Netherlands; 6 Department of Urology, Maastricht University Medical Centre+ Maastricht Netherlands; 7 School for Mental Health and Neuroscience (MeHNS), Maastricht University Maastricht Netherlands; 8 Maastricht University Maastricht Netherlands; 9 Department of Clinical Epidemiology, VieCuri Medical Centre Venlo Netherlands; 10 Department of Gynaecology and Obstetrics, Máxima Medical Centre Veldhoven Netherlands; 11 Department of Psychiatry and Psychology, Maastricht University Medical Centre+ Maastricht Netherlands

**Keywords:** endometriosis, pelvic pain, positive affect, negative affect, patient-reported outcome measure, focus groups, experience sampling method, momentary symptom assessment, mobile phone

## Abstract

**Background:**

Symptoms related to endometriosis have a significant impact on the quality of life, and symptoms often recur. The experience sampling method (ESM), a digital questioning method characterized by randomly repeated momentary assessments, has several advantages over traditionally used measurements, including the ability to assess the temporal relationship between variables such as physical, mental, and social factors.

**Objective:**

The aim of this study is to develop an ESM tool for patients with endometriosis to accurately measure symptoms and their course over time, allowing for personalized treatment and adequate monitoring of treatment efficacy in individual patients.

**Methods:**

On the basis of international guidelines, items from validated questionnaires were selected through a literature review and during focus groups and multidisciplinary expert meetings. Data analysis was conducted using ATLAS.ti (ATLAS.ti Scientific Software Development GmbH). The feasibility and usability of the newly developed momentary assessment tool were tested for 28 consecutive days in 5 patients with endometriosis-related pain symptoms.

**Results:**

Momentary assessment items contained questions concerning endometriosis symptoms, general somatic symptoms, psychological symptoms, contextual information, and the use of food and medication. A morning questionnaire on sleep and sexuality was included. In a pilot study, the patients considered the tool easy to use but time consuming. The average compliance rate of momentary assessments was 37.8% (106/280), with the highest completion rate during the first week (39/70, 56%). Therefore, it is advisable to use the ESM for a maximum of 7 days.

**Conclusions:**

A new digital tool for endometriosis symptom assessment was developed using the ESM, which may help overcome the limitations of current retrospective questionnaires. After validation and testing, future studies will be planned to evaluate the use of this tool in a clinical setting in order to propose a personalized treatment plan for women with endometriosis.

## Introduction

### Background

Endometriosis is defined as an estrogen-dependent condition involving the endometrium-like tissue outside the uterus [1]. It is estimated to be prevalent among approximately 10% in women of reproductive age and up to 50% in women with chronic pelvic pain (CPP) or fertility problems [2,3]. Dysmenorrhea, CPP, dyspareunia, fatigue, and infertility are the leading symptoms [4,5], which have a significant social and psychological impact, decreasing the quality of life of the patients [6-8]. Furthermore, the annual economic burden of women with endometriosis in European countries is high and similar to that of other chronic conditions [8]. The severity of the disease, as well as pelvic pain, infertility, and a higher number of years since diagnosis, are associated with higher costs of societal relevance given that these symptoms affect physical, mental, sexual, and social well-being, as well as work productivity [8-10].

Endometriosis is currently managed by surgical or medical interventions; however, approximately 50% of women have recurrent symptoms over a period of 5 years [11]. Moreover, the extent of endometriosis is not directly related to the degree of the symptoms [12], which suggests that the perception of symptoms may also be influenced by psychological and emotional distress [13-15].

### Objective

Stratified and more individualized therapeutic approaches are needed to maximize treatment efficacy and improve physical, mental, sexual, and social well-being [8-10,16]. To do so, a reliable assessment of endometriosis-related symptoms is essential. Current guidelines for symptom assessment in patients with endometriosis include the recommendations of the Initiative on Methods, Measurement, and Pain Assessment in Clinical Trials [17] and the American Society for Reproductive Medicine [18]. The former has made recommendations for clinical outcomes in pain trials [17], including pain measured in 0 to 10 scales, physical functioning, emotional functioning, symptoms, and adverse events. For patients with endometriosis, the American Society for Reproductive Medicine [18] recommends daily ratings of pelvic pain, daily ratings of dysmenorrhea, and the Endometriosis Health Profile-30 (EHP-30) [19]. Currently, there is no available assessment tool for all contextual factors that could influence endometriosis complaints, including symptom triggers and overlapping symptoms with other comorbidities. Furthermore, validated questionnaires such as the EHP-30 are retrospective. The experience sampling method (ESM) is an electronic questioning method characterized by randomly repeated self-reports on symptoms, activities, emotions, or other elements of real-time daily life [20]. This momentary assessment method has several advantages, including the ability to assess the temporal relationship between variables, high ecological validity, and highly detailed information on the experiences of the subjects. This method aims to provide self-insight, personalized treatment approaches, and adequate monitoring of the effectiveness of these treatments in individual patients. Usually, this method is made available by the use of a mobile app [20-22].

Following the previous development of an ESM tool for psychiatric conditions [20], irritable bowel syndrome (IBS) [22-24], functional dyspepsia [25], and overactive bladder syndrome [26], we aimed to develop an ESM assessment tool for patients with endometriosis.

## Methods

### Overview

This study was conducted between August 2018 and September 2019 and consisted of 5 consecutive phases: initial item selection, focus group interviews to consider input from the patients, critical evaluation through expert meetings, development of the smartphone app, and a pilot study to evaluate feasibility and usability. This study was approved by the Institutional Review Board Ethics Committee of the Maastricht University Medical Centre (MUMC+), Maastricht, the Netherlands (Ref 2018-0674; 2019-1069), and the Máxima Medical Centre, Veldhoven, the Netherlands (Ref 18.122; L19.048).

### Phase I: Question Selection

In agreement with the guidelines of the Food and Drug Administration on patient-reported outcome measure (PROM) development, item selection for the questionnaire started with an initial draft on the basis of the literature of validated outcome measures [27,28]. ESM-specific items concerning psychological, social, and environmental factors were derived from previous ESM validation studies [20-23]. Disease-specific items concerning the quality of life, affective symptoms, and disease-specific symptoms were derived from validated retrospective questionnaires (the Short Form-36, EHP-30, European Quality of Life-5 Dimensions, Generalized Anxiety Disorder-7, Patient Health Questionnaire-9, and Gastrointestinal Symptom Rating Scale-IBS). A list was created with all potentially relevant items from these questionnaires. The phrasing of the items was adjusted to conform to the momentary aspects of ESM assessments. The complete list of items was discussed with a multidisciplinary expert team consisting of gynecologists, endometriosis experts, urologists, a psychiatrist, a gastroenterologist, and a representative of the Dutch endometriosis patient organization. All the items were discussed for potential relevance. In addition, the experts were asked in an open discussion whether there were any relevant items missing according to their field of expertise.

### Phase II: Focus Groups

#### Focus Group Recruitment

Premenopausal patients with endometriosis (diagnosed by physical examination and imaging techniques or laparoscopy) aged ≥18 years were recruited by gynecologists from the ward of the outpatient gynecology department at the MUMC+ or the Máxima Medical Center. Furthermore, patients were recruited through advertisements on the Dutch endometriosis foundation website. Pregnant women and patients with any organic explanation for CPP besides endometriosis were not eligible for participation. Furthermore, participants had to be able to speak and understand written Dutch, as the focus groups were conducted in this language. Written informed consent was obtained from all participants before the study.

#### Focus Group Organization

The focus groups were conducted according to the international PROM development guidelines [27] and the literature on focus group interviews [28]. For each focus group, 6 to 10 patients were invited, and 90-minute sessions were scheduled. The focus groups were conducted in 2 meeting phases according to the focus group guidelines [28], with the guidance of a moderator (EB) and at least one assistant moderator (AL, MP). In the first meeting phase, an open discussion, the participants were instructed to bring forward every item they considered essential for use in a real-time symptom assessment tool. In the second meeting phase, all items derived from the initial draft instrument were discussed in a structured manner. The patients could confirm or criticize the item value for momentary assessments and discussed the phrasing of the questions and the answer options. The focus groups were scheduled one meeting by one until saturation of input was reached, that is, the moment that the meetings no longer contributed any new items or information [25,26].

#### Statistical Analysis

The focus group discussions were voice-recorded and transcribed (JM). Data were qualitatively reviewed and systematically analyzed using ATLAS.ti software (ATLAS.ti Scientific Software Development GmbH; workbench for the qualitative analysis of large bodies of data, eg, textual, audio, and video). Each item was grouped by domain, and all domain items were clustered. When synonyms of items were used, the most frequently mentioned item was selected for the questionnaire.

### Phase III: Expert Meeting

A final meeting with a multidisciplinary expert team (*Phase I: Question Selection*) was arranged to select the items to be used in the final questionnaire. The primary goal of the expert meeting was to critically discuss and convert the findings from the focus groups to generate applicable questions for clinical practice. A second goal was to shorten the list of ESM items to minimize response fatigue and, therefore, noncompliance of patients. All items that were included after the ATLAS.ti analysis of the focus group data were discussed for relevance until a majority was reached. In addition, the experts were asked in an open discussion whether there were any relevant items missing according to their field of expertise (ie, urology, gastroenterology, psychiatry, and gynecology).

### Phase IV: Development of a Smartphone App

The smartphone app MEASuRE (Maastricht Electronic Abdominal Symptom Reporting) was previously created by MEMIC, the center for data and information management at the Faculty of Health, Medicine, and Life Sciences of the Maastricht University and the MUMC+. The app can measure real-time experiences in daily life using the concept of ESM. MEASuRE has been described in previous research and has been adjusted for patients with endometriosis using the questions that were selected in the final expert meeting [22-26].

### Phase V: Pilot Study

The usability of the MEASuRE app has been thoroughly tested in patients with IBS. However, as we adapted the questions to an endometriosis-specific tool, we decided to conduct a pilot study with 5 patients with endometriosis to test the feasibility and usability of these changes to the tool. Given that endometriosis symptoms fluctuate during the menstrual cycle, we aimed to test whether collecting ESM data for 28 consecutive days was feasible [4,10]. Premenopausal women aged at least 18 years and diagnosed with endometriosis were recruited via the ward of the outpatient gynecology department of the MUMC+ or the Máxima Medical Center. The inclusion and exclusion criteria were similar to those in phase II, and written informed consent was obtained before participation. During the study period, ESM assessments were conducted on the patients’ smartphones using the MEASuRE app. Because the sampling procedure should cover a range of waking hours and activities, the momentary assessments started after 7:30 AM and finished before 10:30 PM. The app sent out a notification at 10 random moments during the day, each within a 90-minute time frame, after which the patients could complete the identical electronic self-reports. To minimize the extent to which data were influenced by retrospective biases, the participants had to respond to the notification within the requested time frame (10 minutes). After this period, which has also been described in other studies [20,29], it was no longer possible to start the assessment. Past research has typically used 5 to 10 assessments per day to measure real-time experiences in daily life [29,30]. As missing entries were expected, we also analyzed the rates of compliance of at least 3 out of 10 assessments each day. The participants were called on the second study day to check for technical difficulties and to ensure that the questions were clear. The patients were called and interviewed after 2 weeks and at the end of the pilot study to collect feedback concerning the logistics, usability, and content of the questionnaire.

## Results

### Phase I: Question Selection

**Figure 1:**
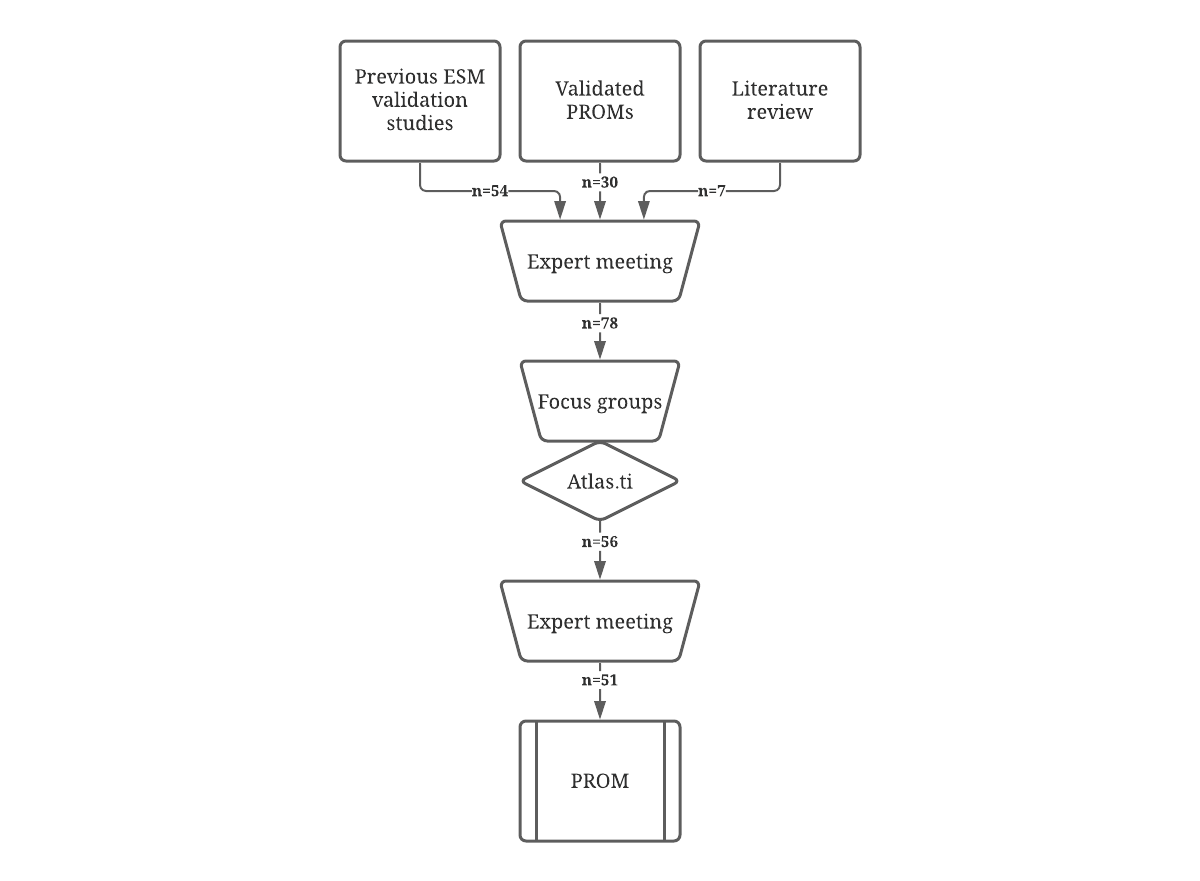
Process of patient-reported outcome measure development. ESM: experienced sampling method; PROM: patient reported outcome measure.

Figure 1 systematically describes the development of a momentary PROM. During question selection, 54 items concerning psychological, social, and environmental factors were derived from questions used in previous ESM validation studies [20-23], whereas 30 items were derived from validated retrospective questionnaires (the Short Form-36, EHP-30, European Quality of Life-5 Dimensions, Generalized Anxiety Disorder-7, Patient Health Questionnaire-9, and Gastrointestinal Symptom Rating Scale-IBS) and made suitable for momentary assessment. Seven questions regarding physical and endometriosis-specific symptoms were added through a clinical literature search [1-5]. During the expert meeting, 13 items were excluded on the basis of relevance. Validated scales such as the Bristol Stool Chart (used in the ESM tool for patients with IBS) and a urological urgency scale were added to make it possible to compare data from patients with endometriosis and patients with other chronic abdominal pain [31,32]. A total of 78 ESM questions were selected concerning different domains: endometriosis-specific symptoms, general somatic symptoms, sleep, sexuality, mood and psychological factors, social and contextual factors, and use of nutrition and medication.

### Phase II: Focus Groups

#### Overview

The characteristics of the women who participated in the focus group meetings are summarized in Table 1. A total of 19 patients initially agreed to participate in the focus groups; however, only 14 were present. The reasons for cancelation were illness (n=2), other plans (n=1), and family-related issues (n=1). One patient did not report any reason for the cancelation. The age of the participants ranged from 23 to 41 years. Saturation of input was reached after 3 focus groups. After the ATLAS.ti analysis of focus group data, the questionnaire comprised 56 items.

**Table 1:** Baseline characteristics.

Variable				Study group		
				Focus groups (n=14)		Pilot study (n=5)
**Sociodemographic**						
	Age (years), mean (SD)			37.1 (6.7)		35.6 (5.6)
	**Level of education, n (%)**					
		High school	1 (7)		0 (0)	
		College or university	13 (93)		5 (100)	
	**Occupational status, n (%)**					
		Student	1 (7)		0 (0)	
		Unemployed	3 (21)		2 (40)	
		Employed	10 (72)		3 (60)	
	**Relationship status, n (%)**					
		Single	1 (7)		1 (20)	
		In relationship	13 (93)		4 (80)	
**Anthropometric**						
	BMI (kg/m^2^), mean (SD)			27.6 (4.7)		26.5 (5.7)
**Medical, n (%)**						
	**Use of hormonal medication**			12 (86)		4 (80)
		Oral contraceptives	4 (29)		2 (40)	
		Mirena IUD^a^	4 (29)		0 (0)	
		Progestins	1 (7)		0 (0)	
		GnRH^b^	4 (29)		2 (40)	
	Regular use of pain medication			11 (79)		5 (100)
	Surgery for endometriosis			11 (79)		3 (60)
	Infertility			5 (36)		1 (20)
	Use of psychiatric medication			2 (14)		0 (0)
	Traumatic life event in past			3 (21)		1 (20)

^a^IUD: intrauterine device.

^b^GnRH: gonadotrophin-releasing hormone.

#### Morning Questionnaire

Women with endometriosis and deep dyspareunia have been found to have lower sexual quality of life, presenting with impaired sexual functioning and decreased satisfaction, which, in turn, can negatively affect personal relationships [33]. Questions concerning sexual activity or avoidance were adapted from the modular dimension *Sexual intercourse* of the EHP-30. Furthermore, the patients considered questions regarding sleep relevant to the general state of well-being. These questions were added to the morning questionnaire, as it was considered unnecessary to assess these items repeatedly during the day [34].

#### Momentary Assessments

Most of the answer options were presented in the numeric rating scale from 0 to 10. However, some questions had answer options on a scale of −5 to +5. The list of questions was shortened by creating subquestions in the case of positive answers. In this matter, questions regarding sexual intercourse, urination, and defecation were asked retrospectively to check whether or not they occurred. If complaints arose, the follow-up questions were asked. The patients stated that the extent of vaginal blood loss was an important issue; however, they also noted that, in the case of absence of a menstrual cycle or after hysterectomy, they did not like to answer any questions regarding blood loss. This was solved by creating a one-off questionnaire on the menstrual cycle after downloading the app. The general somatic questions concerned symptoms as part of a psychosomatic syndrome or caused by the side effects of medication. Questions regarding psychological components were added. These questions concerned both negative and positive affect [20,35,36]. Social and contextual items were added because they could influence physical and emotional well-being and, therefore, the severity of the complaints [20,37]. Questions regarding food intake, use of pain medication, and alcohol consumption were considered essential for influencing pain symptoms or general well-being.

### Phase III: Expert meeting

During the final expert meeting, 6 items were excluded and 1 item was added. The question “How many times did you wake up last night?” was excluded on the basis of relevance, as the quality of sleep and the reason for waking up had already been assessed. In the psychological items, the questions *I feel lonely* and *I feel insecure* were excluded to shorten the list. Furthermore, the ATLAS.ti analysis revealed that these items were mentioned less frequently by patients. Three questions with synonyms regarding energy level (feeling tired, feeling lifeless, and feeling energetic) were adapted to 1 question. The final questionnaire consisted of 51 items (Figure 1). The domains defined during the question selection phase were retained. The number of ESM items varied depending on the answers given by the patients. A morning questionnaire comprised a minimum of 4 and a maximum of 7 questions and included information about sleep and sexuality. Momentary assessments regarding the remaining domains comprised a minimum of 31 and a maximum of 42 items. Table 2 shows the number of questions per category. Two questions were added to a one-off questionnaire on the menstrual cycles of the patients.

**Table 2:** Number of experience sampling method (ESM) questions per category.

Category			Maximum number of ESM questions
**One-off questionnaire**			
	Menstrual cycle	2	
**Morning questionnaire**			
	Sleep	4	
	Sexuality	3	
**Momentary assessment**			
	Endometriosis-specific symptoms	15	
	General somatic symptoms	7	
	Mood and psychological factors	7	
	Social and contextual factors	8	
	Use of nutrition and medication	5	

### Phase IV: Development of a Smartphone App

The final questionnaire that was built into the smartphone app MEASuRE is listed in English in Multimedia Appendix 1. This questionnaire was originally created in Dutch and was officially translated by Medilingua translations; however, it has not yet been validated in English.

### Phase V: Pilot Study

#### Feasibility and Compliance

**Figure 2:**
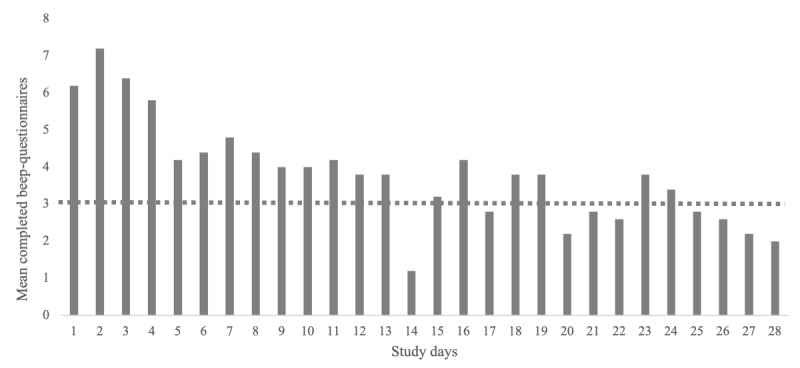
Mean completed momentary assessments per study day. The horizontal dotted line represents the minimum of 3 completed questionnaires per day that is needed for a reliable analysis.

The characteristics of the women who participated in the pilot study are summarized in Table 1. The morning questionnaire took an average of 22 seconds to complete (range 11-44 seconds), and the momentary assessments took an average of 3 minutes and 2 seconds to complete (range of 72-255 seconds). The average completion rate for the morning questionnaires was 81% (23/28 study days). The average response rate for all momentary assessments was 37.86% (530/1400 questionnaires), with a range of 6.1% (17/280) to 56.1% (157/280) between patients. The average completion rate for a minimum of 3 questionnaires was 68% (19/28 study days). The response rate was highest during the first week of the pilot study, on average, 56% (39/70) of questionnaires, with a range of 21% (15/70) to 79% (55/70) between patients. The first week was the only week in which all participants completed at least 3 questionnaires on each study day. Figure 2 shows a histogram with the mean number of completed beep questionnaires (momentary assessments) per study day. In total, 0.79% (11/1400) of the momentary assessments were started but not completed.

#### Interview

During interviews, the patients noted that the app was easy to use and that the questions were clearly defined, although some suggestions were made for clarifications or answer options. Table 3 shows the results of the interviews with patients and includes advantages and limitations. Recommendations from these patients were also included, and their recommendations concerning the content were added to the final questionnaire (Multimedia Appendix 1). There were no particular questions that the patients did not want to answer, such as questions concerning sexuality.

**Table 3:** Advantages, limitations, and recommendations mentioned by the patients that completed the pilot study (n=5).

Theme	Advantages	Limitations	Recommendations
Usability	“Easy to use.” “Completing a questionnaire was getting easier and faster over time.”	“Loading of the questionnaire was slow in case of a bad internet connection.”	Measure period for a maximum of 7 days: “During a menstrual period” (n=1) “Let the patient decide when to start measuring (when the complaints are highest).” (n=3) “Measure period for one month to be sure that one whole menstrual cycle is included. Use less questionnaires per day to improve compliance.” (n=1)
Content	“Very complete.” “The questions are very clear.” “Only by filling in my symptoms and activities at the same time makes me aware of a symptom pattern.” “This is the only questionnaire I know that also contains bowel and bladder symptoms.”	See recommendations for missing items according to the pilot study participants.	“The option I have pain while laying down is missing.” “I am together with my pet.” “The activity: taking care of my children/family.” “Add LEFT (L) and RIGHT (R) to the abdominal pain figure.”
Compliance	“Timing ten times a day is good because then I don’t feel guilty when I miss a questionnaire.”	“Work makes it difficult to complete the questionnaires.” “Social activities make it difficult to complete the questionnaire.” “Less motivation to fill in questionnaires when I don’t have any somatic complaints.” “Less motivation to fill in questionnaires when I am feeling down.”	“Completing 5-7 assessments per day is feasible.” (n=3) “Completing 4-5 assessments per day is feasible.” (n=2)

## Discussion

### Overview

Following the development of an ESM tool specific to psychiatric conditions [20] and gastrointestinal and urological disorders, such as IBS, functional dyspepsia, or overactive bladder syndrome [22-26], we developed a modern assessment tool for patients with endometriosis. This new tool was developed according to the international guidelines on PROM development and comprised 5 phases: a selection of items on the basis of a literature review, a focus group study, expert meetings, the development of an electronic PROM using a smartphone app, and testing of the usability and feasibility with a pilot study. During interviews, the patients noted that the app was easy to use and that the questions were clearly defined. During our pilot study, only 0.79% (11/1400) of all momentary assessments were started but not completed, indicating that the assessments were easy to complete and not too time-consuming. However, completing up to 10 momentary assessments each day was considered time-consuming and caused response fatigue and noncompliance. During a study period of 28 days, most assessments were completed during the first week (39/70, 56%, in the first week vs on average 106/280, 38%, during the total study period). Compared with other ESM studies, this compliance rate is relatively low, as meta-analyses have shown completion rates of 82% to 85% [38,39]. However, comparing data with other ESM studies is difficult because the absence of methodological guidelines related to the use of this method has resulted in a large heterogeneity of designs [39], and compliance rates have not been reported in approximately half of the studies [40]. For better compliance, fewer study days, less assessments per day, and fewer items per assessment are advised [38,40]. In addition, as previous ESM studies recommend at least 3 completed questionnaires per day for a reliable analysis, which occurred consistently only during the first week of this study, we recommend using the ESM for a maximum of 7 days [41]. However, as endometriosis can fluctuate during the menstrual cycle, assessing patients for 4 weeks could add valuable information and might be considered with fewer assessments per day.

### Strengths and Limitations of the ESM

The ESM has several advantages over traditionally used assessment tools, including the ability to evaluate the temporal relationship between variables, high ecological validity, and highly detailed information on the experience of the subject. Furthermore, the ESM allows for a prospective, individualized within‐person approach to symptoms and symptom formation and to treatment outcome, which contrasts with the *average patient* approach of traditional evidence-based practice [37,41]. Self-reports across multiple days and among various participants provide profound and comprehensive insights into the disease course and treatment efficacy. On the basis of this, the ESM may also provide clues for behavioral interventions, adding value to fragmented monodisciplinary treatment, which remains refractory to responsiveness.

A limitation of the ESM is that it is perceived as time-consuming and requires considerable motivation on the part of the patient. Therefore, assessments are ideally kept as brief as possible. Furthermore, assessments several days in a row could encourage rumination. Thus, on the basis of the recommendations of previous studies using ESM, we suggest limiting the assessment period to 7 days and adding items concerning positive affect [20,35,36]. Another concern is selection bias. Not all patients are willing to participate or comply with study protocols using ESM, and participation could be affected by motivation for change in treatment. However, previous research has shown that this method is feasible for a wide variety of patients [42,43].

### Strengths and Limitations of PROM Development

Given that the questions in this new tool are derived from validated questionnaires, this ESM tool designed for use in patients with endometriosis is comparable to validated retrospective PROMs. The use of patient focus groups according to the international guidelines on PROM development strengthens the validity of the questionnaire. A limitation of our focus group study was the limited number of patients who participated. Although 19 patients agreed to participate, only 14 were included in the 3 focus groups. Ideally, 6 to 10 participants were scheduled for each focus group. Most importantly, saturation of input was reached. During the pilot study, a few recommendations were made regarding the content, and these were added to the final questionnaire (Multimedia Appendix 1).

### Future Study Perspectives

This paper comprises the development (part I) of a new PROM for women with endometriosis, with the ability to assess symptoms in real time. The validation stage (part II) will involve testing the psychometric properties of this newly developed tool. A 7-day validation study will be conducted to assess content validity and to investigate the association with potential triggers of physical symptoms, such as psychological, social, and contextual factors. In the planned validation study, 25 patients with endometriosis with CPP at least 1 day per week on average will be included. By letting patients start measuring at random moments, we expect to collect enough data from different menstrual cycle phases and that there will be sufficient data after the use of ESM in 7 consecutive days. Data from this newly developed ESM tool will be compared with frequently used validated (retrospective) outcome measures such as the EHP-30 questionnaire and end-of-day and end-of-week retrospective pain scores. After validation and testing, future studies will be planned to evaluate the use of this tool in a clinical setting in order to propose a personalized treatment plan.

In conclusion, in agreement with the international guidelines, we developed a PROM for real-time symptom assessment in women with endometriosis. This new electronic tool consists of a morning questionnaire and momentary assessments with questions regarding physical, mental, sexual, and social well-being. This tool was considered easy to use and may help overcome the limitations of existing retrospective questionnaires. To minimize noncompliance, it is advised to use this tool for a maximum of 7 days.

## Appendix

Multimedia Appendix 1 Set of questions for the endometriosis-specific experience sampling method–patient-reported outcome measure after focus groups, expert meetings, and pilot study.

## Abbreviations

CPP: chronic pelvic pain
EHP-30: Endometriosis Health Profile-30
ESM: experience sampling method
IBS: irritable bowel syndrome
MEASuRE: Maastricht Electronic Abdominal Symptom Reporting
MUMC+: Maastricht University Medical Centre
PROM: patient-reported outcome measure
