# Supplementary material for: Patient-Reported Outcome Measure for Real-time Symptom Assessment in Women With Endometriosis: Focus Group Study
Source: JMIR Form Res. 2021 Dec 3;5(12):e28782. doi: 10.2196/28782 (PMC8686475; doi:10.2196/28782)
Supplement: Multimedia Appendix 1 [file formative_v5i12e28782_app1.docx]

| *Table S1. Set of questions (q) for the endometriosis-specific ESM-PROM after focus groups, expert meeting, and pilot study.* | |
| --- | --- |
| One-off questionnaire | **Answer options** |
| 1. The following applies to me: | I have regular periods, My periods are irregular, I never experience blood loss  ** in case of regular or irregular periods, q1.1 is asked.  ** in case of never experiencing blood loss, q3 (endometriosis specific symptoms) is not asked.* |
| 1.1 How many days ago did your most recent period start? | 0 t/m 31 days. |
| Morning questionnaire |  |
| Sleep | **Answer options** |
| 1. I slept well | 0 (not at all)—10 (very much so) |
| 2. I had trouble falling asleep. | Yes – No |
| 3. I woke up during the night. | Yes – No ** in case of “Yes”, q3.1 is asked.* |
| - 1. I woke up because of… | An urge to urinate, abdominal pain, worrying, something else/no apparent cause. |
| Sexuality | **Answer options** |
| 4. I had sexual intercourse. | Yes – No ** in case of “Yes”, q4.2 is asked. ** in case of “No”, q4.1 is asked.* |
| 4.1 I avoided sexual intercourse because of pain symptoms. | Yes - No |
| 4.2 I suffered from pain during or after sexual intercourse. | 0 (no pain)—10 (a lot of pain) ** in case of “1 or more”, q4.3 is asked.* |
| - 1. The pain was … | Superficial vaginal pain, deep vaginal pain, abdominal pain  *~More than one answer option possible.* |
| Momentary assessment |  |
| Endometriosis specific symptoms | **Answer options** |
| 1. I suffer from abdominal pain | 0 (no pain)—10 (a lot of pain) ** in case of “1 or more”, q1.1 is asked.* |
| 1.1 I feel this pain in the following place(s) in my abdomen: | 0 – 9  **R L** 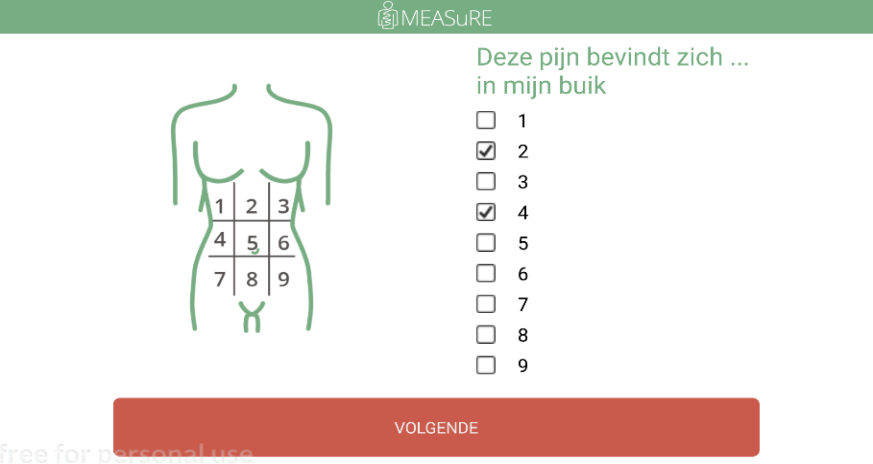 |
| 2. I suffer from pain in my.. | Shoulders, groins, back, anus, legs, other area, none of the above.  *~More than one answer option possible.* |
| - 1. I feel pain when I am… | Walking, sitting, standing, laying down.  *~More than one answer option possible* |
| 3. The vaginal blood loss is as follows: | No bleeding, spotting, light bleeding, moderate bleeding, heavy bleeding. |
| 4. I feel… | Cold (-5) – neutral (0) – very hot/hot flushes(+5) |
| 5. Since the last beep I have urinated... | 0 times, once, twice, 3 times, 4 times, > 4 times. ** In case of “once or more”, q5.1 is asked.* |
| 5.1 When urinating I experienced the following symptoms: | Pain while passing urine, strong urge to urinate, blood loss while passing urine, difficulty passing urine, no symptoms.  ** In case of “strong urge to urinate”, q5.2 is asked.  ~More than one answer option possible* |
| 5.2 How strong was the urge to urinate. | I could put it off for 30 minutes, I could put it off for 10 minutes, I had to urinate instantly/I could not put it off any longer. |
| 6. I feel discomfort due to being bloated | 0(not at all) – 10(very much so) |
| 7. My stomach is distended (swollen). | 0(not at all) – 10(very much so) |
| 8. I feel an urge to defecate. | 0(not at all) – 10(very much so) |
| 9. Since the last beep I have had… bowel movements. | 0, 1, 2, 3, 4, 5, >5  **In case of “1 or more”, q9.1 is asked.* |
| 9.1 The appearance of my stools was as follows: | **Bristol Stool Form Scale**  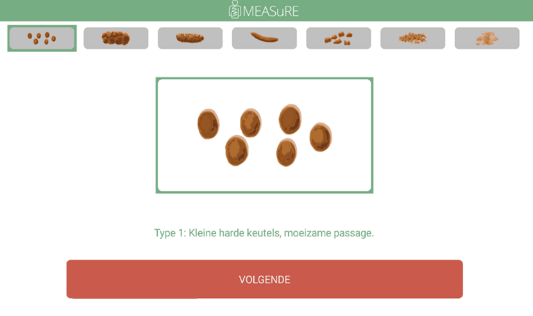 |
| - 1. My bowel movements are painful | 0(not at all) – 10(very much so) |
| - 1. I have rectal bleeding/blood loss. | 0 (not at all)—10 (very much so) |
| General somatic symptoms | **Answer options** |
| 10. I feel dizzy. | 0 (not at all)—10 (very much so) |
| 11. I feel nauseous. | 0 (not at all)—10 (very much so) |
| 12. I have a headache. | 0 (not at all)—10 (very much so) |
| 13. I feel short of breath. | 0 (not at all)—10 (very much so) |
| 14. I have sore muscles and/or joints. | 0 (not at all)—10 (very much so) |
| 15. I feel… | -5 (very tired) – 0 (neutral) – +5 (full of energy) |
| 16. I feel well physically. | 0 (not at all)—10 (very much so) |
| Mood and psychological factors | **Answer options** |
| 17. I feel cheerful. | 0 (not at all)—10 (very much so) |
| 18. I feel dispirited (down). | 0 (not at all)—10 (very much so) |
| 19. I feel emotional. | 0 (not at all)—10 (very much so) |
| 20. I feel stressed. | 0 (not at all)—10 (very much so) |
| 21. I feel relaxed. | 0 (not at all)—10 (very much so) |
| 22. I am worried. | 0 (not at all)—10 (very much so) |
| 23. I feel irritable. | 0 (not at all)—10 (very much so) |
| Social and contextual factors | **Answer options** |
| 24. My current situation is as follows: | I am at home, I am at someone else’s home, I am at work/school, I am in a public place, I am on the move, I am somewhere else. |
| 25. Just before the beep I was… | Resting, at work/school, doing household chores/grocery shopping, taking care of children/providing other informal care, eating/drinking, relaxing/spending time on a hobby, exercising/doing sports, on my way to something, doing something else. |
| 26. I feel uncomfortable/comfortable in this situation. | -5 (very uncomfortable) – 5 (very comfortable) |
| 1. My symptoms are getting in the way of my activities. | 0 (not at all)—10 (very much so)  ** In case of “1 or more”, q27.1 is asked.* |
| 27.1 My symptoms are negatively affecting my… | Household chores, sports/hobbies, work, social activities.  *~More than one answer option possible.* |
| 1. I am currently in the company of… | My partner, children, friends, housemates, co-workers, relatives (who are not part of my household), acquaintances, people I don’t know/others, a big group, one or more pets, no one.  ** In case “no one” is NOT answered, q28.1 is asked.* |
| 28.1 I feel uncomfortable/comfortable in this company. | -5 (very uncomfortable) – 5 (very comfortable) |
| 1. I feel that I need to rest. | 0 (not at all)—10 (very much so) |
| Use of nutrition and medication | **Answer options** |
| 1. Since the last beep I have eaten… | Breakfast, lunch, dinner, a snack/fruit, nothing |
| 1. Since the last beep I have taken/had… | Caffeine (coffee), nicotine (smoking), alcohol, narcotic drugs, painkillers, none of the above.  ** In case of “alcohol”, q31.1 is asked. ** In case of “painkillers”, q31.2 and q31.3 are asked.* |
| 31.1 Since the last beep I have had … units of alcohol. | 0-2, 3-4, > 4 |
| 31.2 Since the last beep I have used painkillers for… | Abdominal pain, something else. |
| 31.3 Since the last beep I have used the following painkiller ... | Paracetamol, an NSAID (ibuprofen, Aleve, naproxen, diclofenac, Arcoxia), tramadol/Zaldiar, an opioid (e.g. oxycodone), homoeopathic preparations, CBD oil (cannabis oil)/ hashish/ THC. |

pling research in individuals with mental illness: reflections and

guidance. Acta Psychiatr Scand. 2011;123(1):12–20.

30. Delespaul P. Assessing schizophrenia in daily life the experience

samplingmethod.UPM,UniversitairePersMaastricht

University; 1995.

Palmier‐ClausJE,Myin‐GermeysI,BarkusE,etal.Experiencesam‐

pling research in individuals with mental illness: reflections and

guidance. Acta Psychiatr Scand. 2011;123(1):12–20.

30. Delespaul P. Assessing schizophrenia in daily life the experience

samplingmethod.UPM,UniversitairePersMaastricht,Maastricht

University; 1995.

Palmier‐ClausJE,Myin‐GermeysI,BarkusE,etal.Experiencesam‐

pling research in individuals with mental illness: reflections and

guidance. Acta Psychiatr Scand. 2011;123(1):12–20.

30. Delespaul P. Assessing schizophrenia in daily life the experience

samplingmethod.UPM,UniversitairePersMaastricht,Maastricht

University; 1995.

Palmier‐ClausJE,Myin‐GermeysI,BarkusE,etal.Experiencesam‐

pling research in individuals with mental illness: reflections and

guidance. Acta Psychiatr Scand. 2011;123(1):12–20.

30. Delespaul P. Assessing schizophrenia in daily life the experience

samplingmethod.UPM,UniversitairePersMaastricht,Maastricht

University; 1995.

Palmier‐ClausJE,Myin‐GermeysI,BarkusE,etal.Experiencesam‐

pling research in individuals with mental illness: reflections and

guidance. Acta Psychiatr Scand. 2011;123(1):12–20.

30. Delespaul P. Assessing schizophrenia in daily life the experience

samplingmethod.UPM,UniversitairePersMaastricht,Maastricht

University; 1995.

Palmier‐ClausJE,Myin‐GermeysI,BarkusE,etal.Experiencesam‐

pling research in individuals with mental illness: reflections and

guidance. Acta Psychiatr Scand. 2011;123(1):12–20.

30. Delespaul P. Assessing schizophrenia in daily life the experience

samplingmethod.UPM,UniversitairePersMaastricht,Maastricht

University; 1995.

Palmier‐ClausJE,Myin‐GermeysI,BarkusE,etal.Experiencesam‐

pling research in individuals with mental illness: reflections and

guidance. Acta Psychiatr Scand. 2011;123(1):12–20.

30. Delespaul P. Assessing schizophrenia in daily life the experience

samplingmethod.UPM,UniversitairePersMaastricht,Maastricht

University; 1995.

Palmier‐ClausJE,Myin‐GermeysI,BarkusE,etal.Experiencesam‐

pling research in individuals with mental illness: reflections and

guidance. Acta Psychiatr Scand. 2011;123(1):12–20.

30. Delespaul P. Assessing schizophrenia in daily life the experience

samplingmethod.UPM,UniversitairePersMaastricht,Maastricht

University; 1995.

Palmier‐ClausJE,Myin‐GermeysI,BarkusE,etal.Experiencesam‐

pling research in individuals with mental illness: reflections and

guidance. Acta Psychiatr Scand. 2011;123(1):12–20.

30. Delespaul P. Assessing schizophrenia in daily life the experience

samplingmethod.UPM,UniversitairePersMaastricht,Maastricht

University; 1995.

Palmier‐ClausJE,Myin‐GermeysI,BarkusE,etal.Experiencesam‐

pling research in individuals with mental illness: reflections and

guidance. Acta Psychiatr Scand. 2011;123(1):12–20.

30. Delespaul P. Assessing schizophrenia in daily life the experience

samplingmethod.UPM,UniversitairePersMaastricht,Maastricht

University; 1995.

Palmier‐ClausJE,Myin‐GermeysI,BarkusE,etal.Experiencesam‐

pling research in individuals with mental illness: reflections and

guidance. Acta Psychiatr Scand. 2011;123(1):12–20.

30. Delespaul P. Assessing schizophrenia in daily life the experience

samplingmethod.UPM,UniversitairePersMaastricht,Maastricht

University; 1995.
